# Automatic treatment planning for VMAT‐based total body irradiation using Eclipse scripting

**DOI:** 10.1002/acm2.13189

**Published:** 2021-02-10

**Authors:** Jose R. Teruel, Sameer Taneja, Paulina E. Galavis, K. Sunshine Osterman, Allison McCarthy, Martha Malin, Naamit K. Gerber, Christine Hitchen, David L. Barbee

**Affiliations:** ^1^ Department of Radiation Oncology NYU Langone Health New York NY USA

**Keywords:** advanced treatment planning, eclipse scripting, plan automation, special procedures, total body irradiation (TBI)

## Abstract

The purpose of this work is to establish an automated approach for a multiple isocenter volumetric arc therapy (VMAT)‐based TBI treatment planning approach. Five anonymized full‐body CT imaging sets were used. A script was developed to automate and standardize the treatment planning process using the Varian Eclipse v15.6 Scripting API. The script generates two treatment plans: a head‐first VMAT‐based plan for upper body coverage using four isocenters and a total of eight full arcs; and a feet‐first AP/PA plan with three isocenters that covers the lower extremities of the patient. PTV was the entire body cropped 5 mm from the patient surface and extended 3 mm into the lungs and kidneys. Two plans were generated for each case: one to a total dose of 1200 cGy in 8 fractions and a second one to a total dose of 1320 cGy in 8 fractions. Plans were calculated using the AAA algorithm and 6 MV photon energy. One plan was created and delivered to an anthropomorphic phantom containing 12 OSLDs for in‐vivo dose verification. For the plans prescribed to 1200 cGy total dose the following dosimetric results were achieved: median PTV V100% = 94.5%; median PTV D98% = 89.9%; median lungs Dmean = 763 cGy; median left kidney Dmean = 1058 cGy; and median right kidney Dmean = 1051 cGy. For the plans prescribed to 1320 cGy total dose the following dosimetric results were achieved: median PTV V100% = 95.0%; median PTV D98% = 88.7%; median lungs Dmean = 798 cGy; median left kidney Dmean = 1059 cGy; and median right kidney Dmean = 1064 cGy. Maximum dose objective was met for all cases. The dose deviation between the treatment planning dose and the dose measured by the OSLDs was within ±4%. In summary, we have demonstrated that scripting can produce high‐quality plans based on predefined dose objectives and can decrease planning time by automatic target and optimization contours generation, plan creation, field and isocenter placement, and optimization objectives setup.

## 1 INTRODUCTION

Total body irradiation (TBI) is a special radiation therapy (RT) procedure in which radiation is administered to the full body of the patient. In combination with chemotherapy, TBI is one of the therapeutic components of conditioning regimens used to condition patients with hematological neoplasms for hematopoietic stem cell transplantation (HCT), primarily those affected by acute myeloid leukemia (AML) and acute lymphoid leukemia (ALL). TBI enhances antineoplastic therapeutic efficacy due to its potential to reach sanctuary sites, such as the testis and the central nervous system (CNS), and provides immunosuppression that prevents bone marrow transplant rejection.^1^, ^2^, ^3^, ^4^ Radiation‐induced interstitial pneumonitis is a major concern for patients undergoing TBI. According to the International Lymphoma Radiation Oncology Group, pneumonitis occurs in about 25% of patients receiving fractionated TBI.^1^

From a physics standpoint, guidelines for administering TBI are outlined in report no. 17 from the AAPM Task Group 29.^5^ Historically, administration of TBI is delivered with the patient at an extended distance (extended SSD), such that the radiation field encompasses the patient's entire body. This technique continues as the standard of practice in most cancer centers performing TBI. Open field treatments used for conventional TBI normally require the use of a beam spoiler to ensure coverage at shallow depths when using high‐energy beams. Compensators are recommended as well in an effort to obtain homogenous dose distributions.^6^, ^7^ However, previous work has demonstrated that conventional hand calculations for TBI without tissue heterogeneity correction result in significant dose underestimation, particularly in the lungs for high‐energy bilateral treatments.^8^

One of the main drawbacks of at‐distance open beam treatments used for TBI is the inability for selective sparing of organs at risk (OARs). Interstitial pneumonitis is the major side effect from high‐dose TBI radiation therapy and can be fatal in some instances.^1^, ^9^, ^10^, ^11^ Additionally, high‐dose TBI treatments can produce late toxicities, such as chronic kidney dysfunction and secondary malignancies.^12^, ^13^, ^14^, ^15^ In order to mitigate TBI‐induced acute and chronic side effects, the use of shielding blocks, particularly for lungs and in some instances for kidneys, is widely accepted in high‐dose TBI treatments.^16^, ^17^, ^18^, ^19^ However, accurate placement of beam modifying devices relative to the patient in treatment position presents challenges due to limitations in image verification, intrafraction patient motion, and reproducible patient setups.

More recently, several alternatives to at‐distance treatment for TBI have been reported. Helical Tomotherapy (Accuray Inc, Sunnyvale, CA) has been employed in single institution studies for TBI and total marrow irradiation (TMI) treatments to obtain higher coverage for sites at high risk of recurrence while sparing major OARs, such as the lungs, liver, and kidneys.^20^, ^21^, ^22^, ^23^ In addition, there is ongoing effort to explore the use and feasibility of volumetric arc therapy (VMAT)‐based TBI treatments in order to obtain a more homogeneous dose distribution, better target coverage, and better sparing of lungs, kidneys, and any other organs with increased risk due to patient comorbidities or previous radiation history.^24^, ^25^, ^26^, ^27^, ^28^

Treatment planning approaches for VMAT‐based TBI are not yet standardized, can be hard to develop, and may vary across institutions. Placement of fields, isocenters, and planning technique is solely based on each institution's individual efforts and experience due to the novelty of the technique and the lack of standardization. The purpose of this work is to establish a fully automated approach for isocenter and treatment field placement as well as dose objectives selection for optimization for VMAT‐based TBI treatment planning. This objective serves two purposes: First, to provide a consistent and standardized planning technique. Second, to ensure consistent shifts at treatment in order to prevent errors when treating multiple isocenters.

## 2 MATERIAL AND METHODS

### 2.A Subjects

Five full body anonymized CT scans from adult patients previously treated at our institution using conventional TBI were used retrospectively. CT images were obtained using a CT SOMATOM scanner (Siemens, Erlangen, Germany). Patients were simulated in the supine position. The protocol employed a 1 cm slice thickness, 500 mm acquisition diameter, and extended field of view (FOV) reconstruction of 650 mm. The use of anonymized retrospective CT scans for dosimetry studies was approved by our Internal Review Board and consent was waived. Additionally, an anthropomorphic body phantom (CIRS, Norfolk, VA) was scanned using the same CT simulator and a 5mm slice thickness. The phantom included all anatomical sections from mid‐thigh to head with no upper or lower extremities.

### 2.B Treatment planning

A script was developed to automate and standardize the treatment planning process. The script was developed in C# programming language using the Varian Eclipse v15.6 Scripting API (Varian Medical Systems, Palo Alto, CA). A summary of the treatment planning tasks that were automated is presented in Supplemental Figure S1. The input of the script is a structure set that must include at a minimum the following contours: body, lungs (including unilateral contours) and kidneys (including unilateral contours). These contours are created manually by the dosimetrist. Additionally, the user origin location needs to be entered prior to running the script. For these retrospective cases, an approximate location at body midline (anterior–posterior), lungs midline (craniocaudal), and sternum (left–right) was employed. For prospective patients to be treated with VMAT‐TBI the user origin location will be determined at simulation and radiopaque ball bearings (bbs) will be placed to allow for user origin placement during treatment planning.

**FIG. 1:**
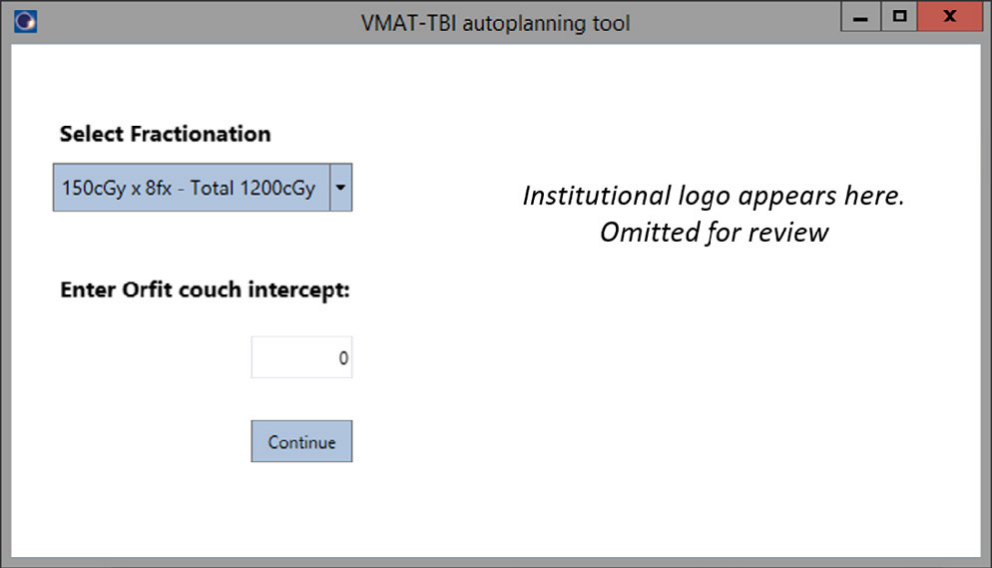
Graphic user interface of the treatment planning tool showing dropdown to select fractionation. Additionally, it requests an input value to calculate all the treatment couch longitudinal values based on the laser intercept at simulation.

Script execution prompts the user to select a fractionation option and provide the intercept of the laser with the couch top longitudinal scale at the user origin location recorded at simulation (Fig. 1). Currently, two options are available for planning: 150 cGy × 8 fractions BID to a total dose of 1200 cGy; and 165 cGy × 8 fractions BID to a total dose of 1320 cGy.

**FIG. 2:**
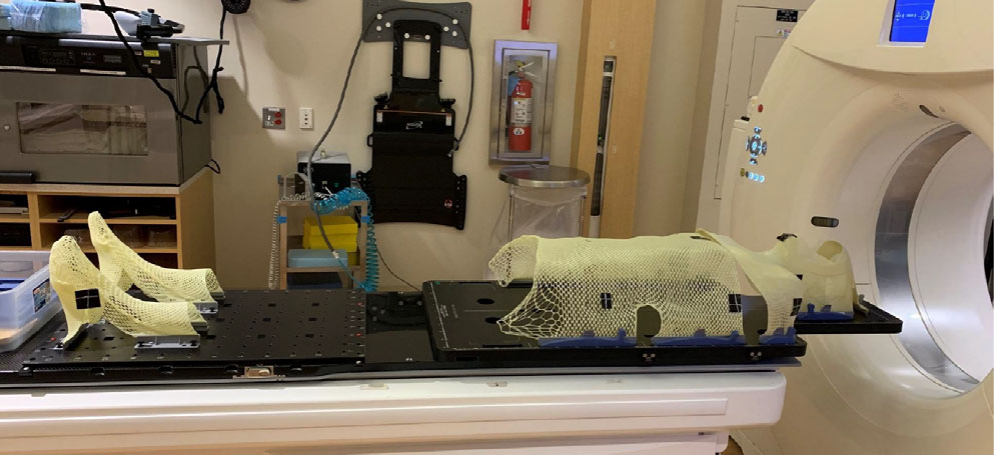
Orfit couch tops and thermoplastic immobilization for VMAT‐based TBI CT simulation. The presented thermoplastic devices were prepared on a healthy volunteer as part of establishing the program. Thermoplastic immobilization for the upper body board have fixed (one location) indexing. Thermoplastic immobilization for each individual foot can be placed in several locations in the board to allow for comfortable immobilization for patients with different heights as well as to control for leg separation.

**FIG. 3:**
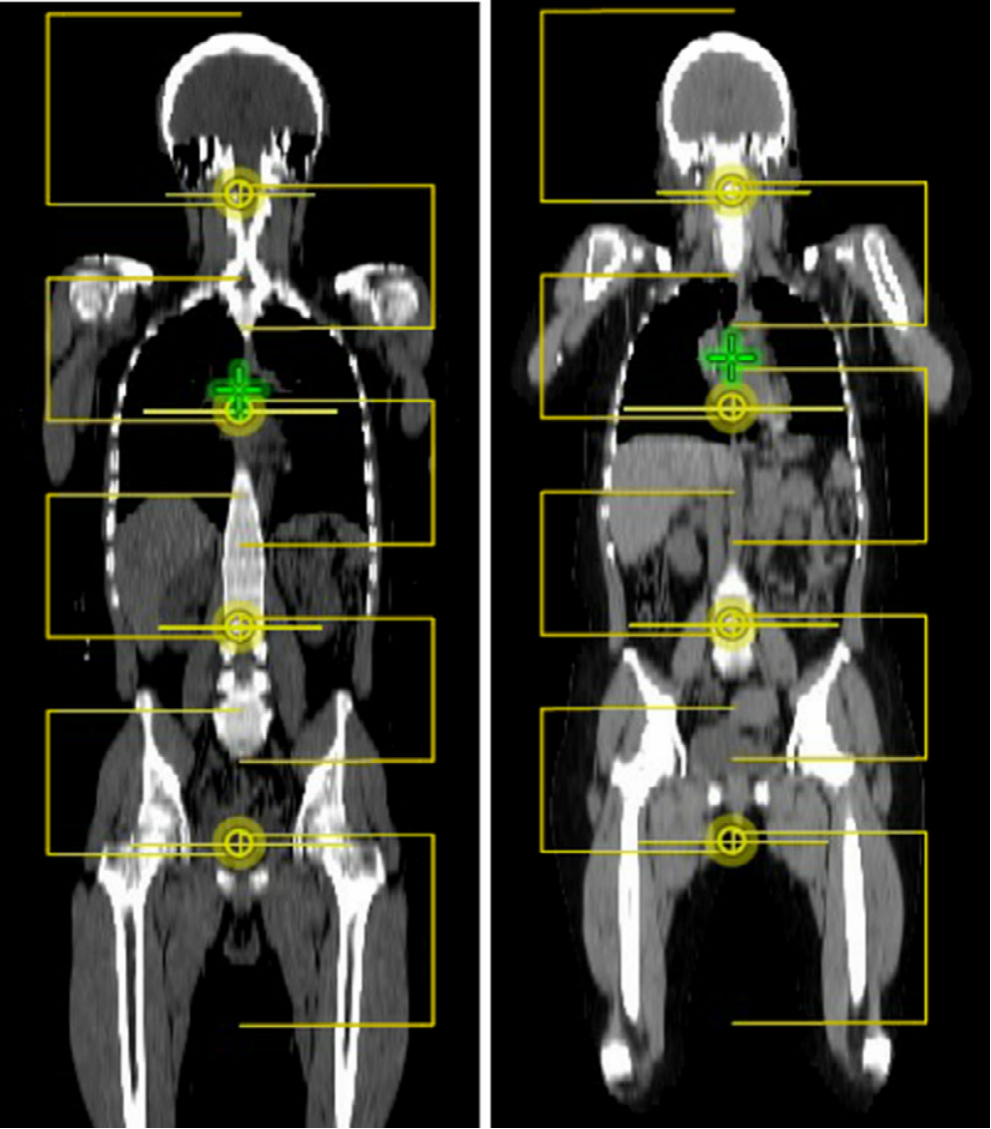
Initial VMAT field arrangement for two cases right after script execution. In both cases the total craniocaudal length covered by the fields remain the same (106 cm). Fields that share an isocenter overlap by 2 cm. Fields that not share an isocenter overlap by 5.3 cm. The final jaw shape is defined during optimization using jaw tracking. Left: Larger patient where maximum coverage of upper body VMAT fields stop mid‐thigh. Right: Shorter patient demonstrating coverage up to the superior aspect of the knee. The superior jaw of the fourth field (chest isocenter, inferior field) in this case is automatically extended 3 cm to compensate for the isocenter location inferiorly in the lungs.

After the planner selects the option according to prescription, the script performs the following tasks automatically. The script creates one treatment course that contains two automatically generated plans, an upper body VMAT plan and a lower body 3D plan. The upper body plan contains four isocenters with two full arcs per isocenter. For the generation of this upper body plan the script uses prior knowledge of the couch indexing and couch longitudinal travel to maximize the area of the patient treated with VMAT. Two couch tops (Orfit Industries, Wijnegem, Belgium) were selected for prospective VMAT‐TBI treatments to provide immobilization required for VMAT delivery and reduce setup variability. The superior couch top provides a fixed location for the head rest with five point indexing for head and neck thermoplastic immobilization mask and four point indexing for chest and abdomen thermoplastic immobilization cast. The inferior couch top is an extremity plate that allows individual indexing of both feet separately. The complete arrangement including couch tops and immobilization masks is presented in Fig. 2. Standardized indexing of the couch tops on top of the linear accelerator couch together with the fixed position of the head rest in the couch tops, allows for a consistent head isocenter location at 87 cm longitudinal (±1 cm tolerance) and a maximum accepted 155 cm longitudinal (±1 cm) for the pelvis isocenter that is built into the script (maximum longitudinal of 160 cm on Varian 6 degrees of freedom couch). The script places equidistant isocenters starting superiorly and includes 2 cm flash from the most superior slice of the body contour. The exact process employed by the script for isocenter placement for the upper body VMAT plan is the following: (a) The script finds the body contour and locates the most superior slice of the contour (top of the head). (b) The script adds 2 cm superiorly to the CT image longitudinal coordinate (to allow for flash). (c) The script sets the first isocenter (head isocenter) 19 cm inferior from the previously calculated longitudinal coordinate. (d) The script creates all subsequent isocenters using the information regarding the couch travel limitation presented above and resulting in an isocenter spacing of 22.7 cm. The treatment fields are set with 90° collimator rotation and jaws are set asymmetrically to obtain an overlap of 2 cm for fields sharing an isocenter, and an overlap of 5.3 cm for arcs of adjacent isocenters. The 2 cm overlap for the fields sharing the chest isocenter can be adjusted automatically by the script, if required, to improve lungs sparing. The script calculates the center slice of the lungs contour in the craniocaudal direction and will increase the jaw openings if the field edge of any of the jaws used to create the MLC based island block is more than 2 cm away from the center of the lungs. An example is presented in Fig. 3. The overlap is defined at the user origin coronal plane. For the inferior half of the patient that cannot be accommodated in the head‐first orientation, the script creates a lower body, feet‐first plan using three equally spaced isocenters. This lower body plan is a 3D anterior–posterior/posterior–anterior (AP/PA) plan with field matching at the isocenter plane. The isocenters for the lower body plan are placed 4 cm posteriorly compared to the upper body plan to account for the smaller anterior–posterior width on the legs compared to the chest and abdomen. Overlap between the lower body plan and upper body plan, as well as flash for the most inferior field is accounted by the script using the following steps: (a) The script finds the most inferior slice of the body contour. (b) The script adds 3 cm inferiorly to the longitudinal location detected in the prior step. (c) The script calculates the distance from this slice to the most inferior slice included in the upper body VMAT plan. (d) The script adds 5 cm to that distance creating overlap between the lower body and the upper body plan. (e) The script splits that distance into three equally spaced isocenters. (f) The script creates two fields (AP/PA) at each isocenter location with symmetric jaws and field matching at the user origin plane.

The script creates four targets: PTV; PTV_Sup; PTV_Inf; and PTV_Sup_Norm. The automatically generated PTVs are defined according to the following definitions:

- PTV is the body contour with a 5 mm margin inside the body surface and extended 3 mm into the lungs and kidneys.
- PTV_Sup is defined as the subsection of the PTV that is covered by the superior VMAT fields.
- PTV_Inf is the subsection of the PTV defined as the PTV minus PTV_Sup.
- PTV_Sup_Norm is defined as the PTV_Sup excluding the region of overlap with the inferior AP/PA fields.

**FIG. 4:**
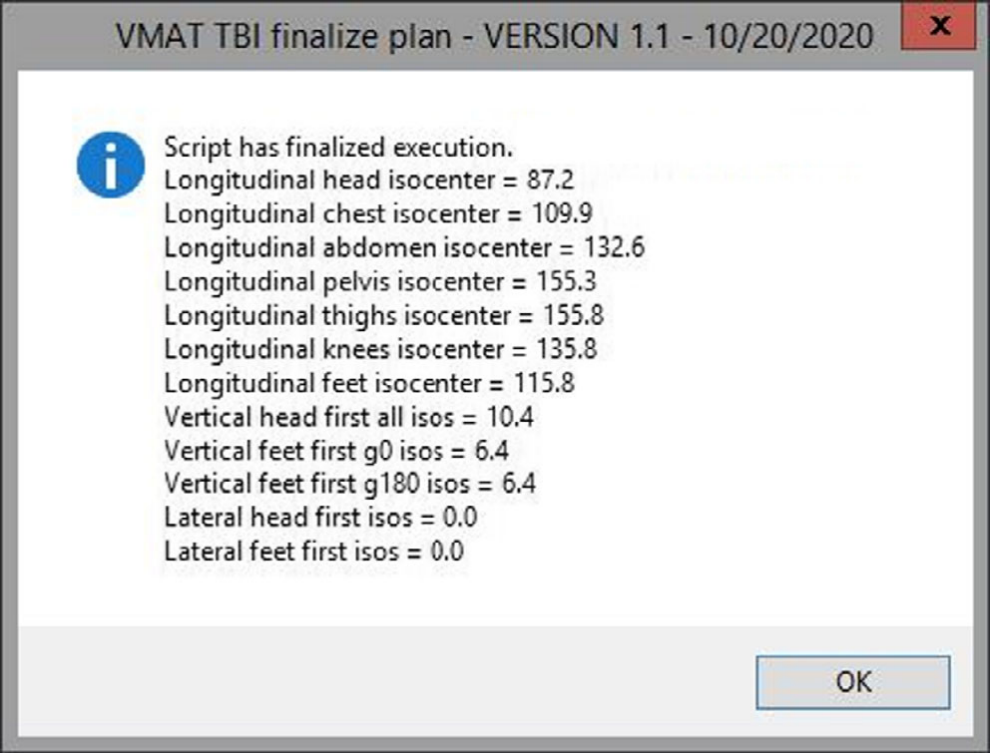
Script output presenting treatment couch values to assist dosimetrist with treatment preparation. The script outputs each isocenter's longitudinal couch value and vertical couch values grouped by plan and field (in case an extended distance is required for the lower body plan) and lateral couch values for each plan. Additionally, these values are exported by the script into a “.csv” file.

Additionally, the script creates eight optimization structures. Optimization structures are defined as the union of each individual VMAT field with the PTV structure. Due to the divergence of the fields, we consider the intercept at the coronal plane of the user origin. These optimization structures are named opt_ptv_X, where X is the beam number of each individual field from the upper body VMAT plan. Therefore, the optimization structures are named consecutively from opt_ptv_1 (union of the PTV and the area covered by first field of the head isocenter) to opt_ptv_8 (union of the PTV and the area covered by the second field of the pelvis isocenter). The script will create all optimization objectives based on the fractionation selected and will load them automatically for optimization. Some additional options, such as aperture shape controller (set to moderate), air cavity correction (set to on), and jaw tracking (set to enabled), are set automatically by the script for the photon optimizer PO v.15.6. Finally, the script uses the user origin location, the couch intercept value entered by the planner in the graphic user interface, and the couch entered in the CT to provide the planner with all couch coordinates pertaining to the created isocenter arrangement. The script presents these values on the screen (Fig. 4), and creates a “.csv” file to be loaded in an in‐house tool employed to assist with imaging and treatment delivery (out of the scope of this report). Additionally, the time required to run the script was compared to the time required to manually perform all the tasks completed by the script.

After script execution, the weights of the inferior 3D plan are manually adjusted to maximize coverage of the inferior target structure (PTV_Inf) while maintaining the maximum dose in that region below 130%. In the next planning step, a second instance of the script is executed to automatically run the optimization of the upper body VMAT plan using the preloaded dose objectives, and automatically calculate the plan after optimization. This second part of the script can be run in standalone mode, therefore, opening an independent instance of Eclipse, saving the progress and closing Eclipse allowing the planner to execute it after hours or overnight.

All treatment plans were created using a Varian TrueBeam machine with Millennium MLC, 2.5 mm optimization grid, 5 mm calculation grid, air cavity correction, and the analytic anisotropic algorithm (AAA). The energy employed was 6 MV photons for all fields with a maximum allowed nominal dose rate of 600 MU/min.

### 2.C Dose objectives

Two levels of planning goals were established for this treatment planning study. Primary goals must be met for every plan and include: target coverage of PTV V100% >90%, target near minimum dose of D98% >85%, and target maximum dose of PTV D2cc <130%. The primary goals for the organs at risk (OARs) include: lung mean dose <800 cGy for 1200 cGy plans or <900 cGy for 1320 cGy plans, individual kidney mean dose <1100 cGy, and maximum dose to any OAR D0.03cc <120%. Secondary goals include the following: target coverage PTV V100% >95%, target near minimum dose of PTV D98% >90%, and a target mean dose <110%. For the OARs, the secondary goal is lung mean dose <800 cGy for 1320 cGy plans.

### 2.D In‐vivo dose verification, QA, and plan uncertainty analysis

For in‐vivo dose verification one treatment plan was created for the CT dataset of the anthropomorphic phantom. This plan was created using the script and following the same rationale as described for the patient dataset with the only difference being the absence of a lower body plan (phantom does not include extremities). The treatment plan was delivered to the phantom previously loaded with optically stimulated luminescence dosimeters (OSLDs). Twelve OSLDs were placed in different key locations including lungs (four OSLDs), kidneys (two OSLDs), soft tissue (two OSLDs), and bone (four OSLDs). OSLDs were placed inside the phantom using drilled OLSD holders provided by the vendor. OLSDs were read using a microSTARii system (Landuaer, Glenwood, IL). The OSLD reader was calibrated following vendor recommendations, daily QA was performed prior to reading OSLDs, and nonpreviously irradiated or annealed nanoDots were employed (quoted uncertainty by the vendor of 5.5%). The dose difference between the recorded dose by the OSLD and the treatment planning system (TPS) dose was evaluated using a normalized dose difference metric defined as:

$$Dosedeviation\left(\%\right)=100\frac{Dose_{\mathrm{OSLD}}‐Dose_{\mathrm{TPS}}}{Dose_{\mathrm{Rx}}}$$

Patient‐specific QA was performed for this plan using three different methods: (a) A dose verification plan was created for a SNC ArcCheck (SunNuclear, Melbourne, FL) diode array device and each field was evaluated separately; (b) A portal dosimetry verification plan was delivered to the electronic portal imager device (EPID) and analyzed using Varian Portal Dosimetry tool for each field; (c) Portal Dosimetry measurements were analyzed using SNC PerFRACTION Fraction 0. Additionally to patient‐specific QA verification using the described three methods for each individual field, the area of overlap between fields not sharing an isocenter was measured and analyzed using SNC ArcCheck.

Finally, we analyzed plan uncertainty for the phantom treatment plan in two ways. First, we evaluated plan uncertainty by applying the same rigid shifts to all isocenters for 5 mm and 10 mm shifts for each individual axis (lateral, vertical, and longitudinal). Second, we evaluated dose profiles at the overlap regions of fields not sharing an isocenter.

## 3 RESULTS

**FIG. 5:**
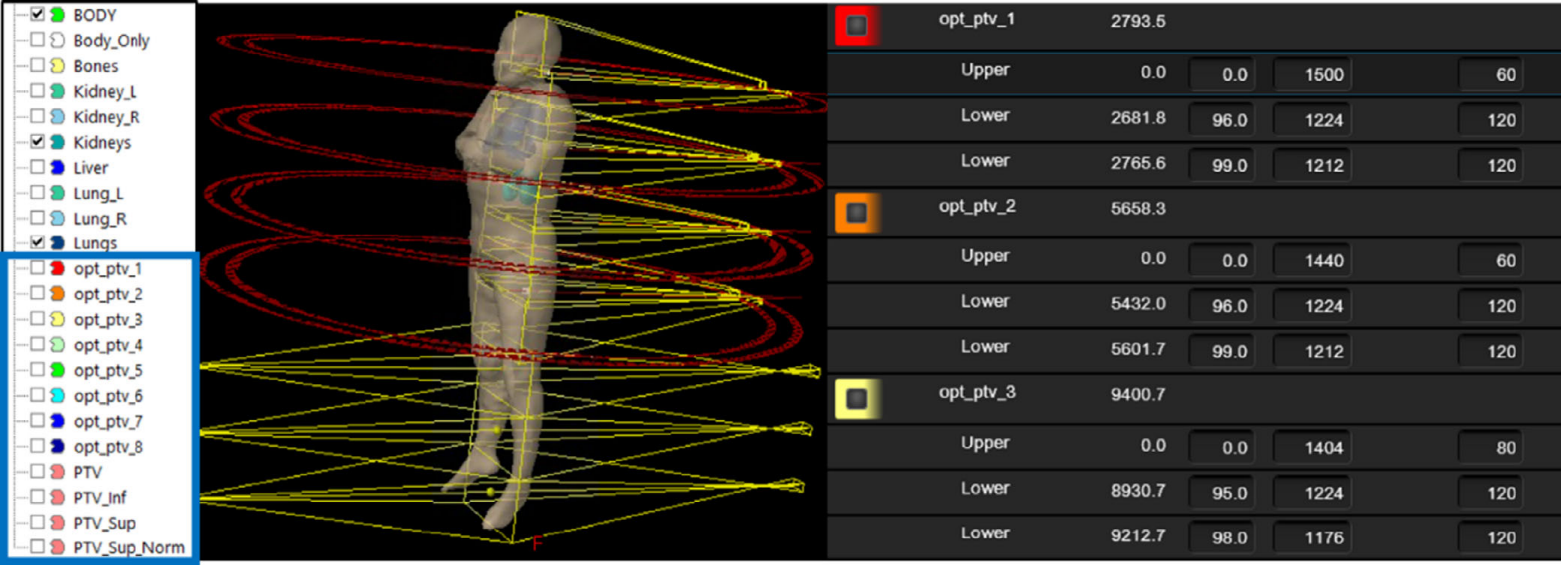
Results after execution of the automatic planning script. Left: Structure set. The structures inside the blue box are automatically generated as described in the Methods section. Middle: Automatic field placement result. Right: Example of some of the optimization objectives loaded by the script.

The result from the script execution is illustrated in Figs. 4 and 5. The total time the initial script takes to run is negligible in terms of planning time (less than 10 s using a Varian workstation). This included course creation, creation of superior and inferior plans, calculation of optimal location of isocenters, placement of inferior plan fields, placement of superior plan fields, creation of four target contours and eight optimization contours, and loading the appropriate optimization template, and calculation of all treatment couch coordinate for each isocenter. The time required to perform all these tasks manually was variable, but it was estimated to comprise approximately 2–3 hrs with the calculation of optimal location of isocenters taking about 20–40 min, the setup of fields for both plans with appropriate overlap and jaw setting about 40–60 min, the creation of all target and optimization contours about 30–50 min, manual calculation of each treatment couch coordinate for 7 isocenters about 1020 min, and the rest of the tasks required prior to optimization taking about 20 min. The time required to perform all the tasks manually considers that they are all completed successfully. However, we found that due to the complexity, even a small error in setting up isocenters or manual field overlap may go unnoticed until after optimization which may result in up to a full day of time savings when using the script for these tasks.

The body habitus volumes of the cases under study ranged between 56599 and 90932 cm^3^ with a height range between 152 and 181 cm, maximum lateral width between 45.4 and 62.2 cm, and lung volume between 1716 and 3260 cm^3^. All measurements were obtained directly from the planning CT and represent the dimension of the patient in simulation position. In Fig. 3, two cases with different patient heights are presented, illustrating how the script prioritizes coverage in the craniocaudal orientation with the upper body VMAT plan.

**FIG. 6:**
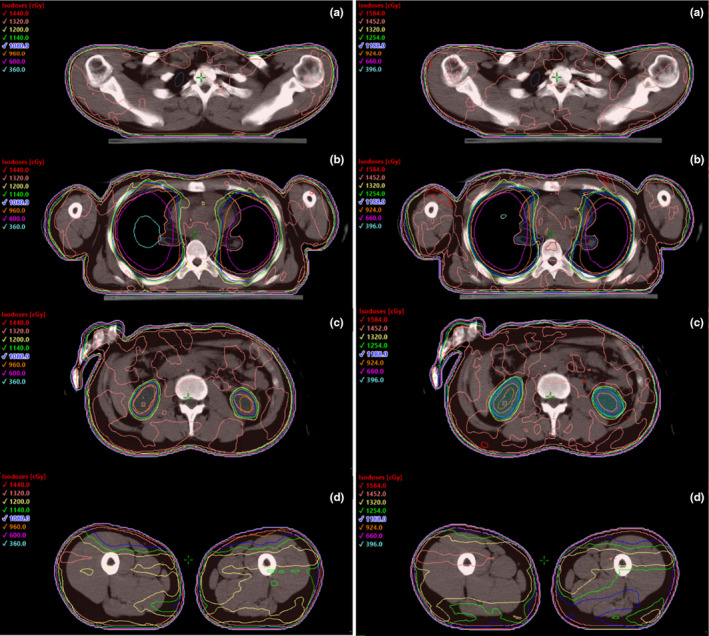
Dosimetric distribution in four axial views. Top to bottom: (a) Area of overlap between two fields with different isocenters (head isocenter and chest isocenter). (b) Slice showing the lungs. (c) Slice showing the kidneys. (d) Area of overlap between upper body VMAT treatment plan and lower body AP/PA treatment plan (AP/PA plan used as base plan for VMAT optimization). Left: Treatment plan for 1200 cGy (150 cGy per fraction). Right: Treatment plan to 1320 cGy (165 cGy per fraction).

All primary dosimetric goals were met for all calculated plans. A summary of the dosimetric results is presented in Table 1 for 1200 cGy total dose plans and in Table 2 for 1320 cGy total dose plans. These results were obtained with only one optimization using the generic goals loaded by the script. For the plans prescribed to 1200 cGy total dose (150 cGy × 8 fractions) the following dosimetric results were achieved: median PTV V100% = 94.5%; median PTV D98% = 89.9%; median lungs Dmean = 763 cGy; median left kidney Dmean = 1058 cGy; and median right kidney Dmean = 1051 cGy. For the plans prescribed to 1320 cGy total dose (165 cGy × 8 fractions) the following dosimetric results were achieved: median PTV V100% = 95.0%; median PTV D98% = 88.7%; median lungs Dmean = 798 cGy; median left kidney Dmean = 1059 cGy; and median right kidney Dmean = 1064 cGy. The isodose coverage at four different relevant levels (lungs, different isocenter field matching, kidneys, superior VMAT, and inferior APPA match region) are presented in Fig. 6 for both treatment plans (1200 cGy and 1320 cGy total dose) for one case.

**TABLE 1:** Dose objectives and results for 1200 cGy total dose plans (150 cGy per fraction) generated using treatment planning script. Median values with minimum and maximum in parentheses. Objectives defined as a range are primary–secondary goals.

Structure	Dose objective	Result
PTV	V100% >90‐95%	94.5% (93.7, 95.2)%
	D98% >85‐90%	89.9% (88.7, 91.3)%
	Dmean <110%	108.6% (106.1, 110.0)%
Lungs	Dmean <800 cGy	763 cGy (729, 783) cGy
	D0.03cc <120%	116.1% (114.4, 118.0)%
Left kidney	Dmean <1100 cGy	1058 cGy (1030, 1066) cGy
	D0.03cc <120%	110.2% (108.4, 112.7)%
Right kidney	Dmean <1100 cGy	1051 cGy (1025, 1083) cGy
	D0.03cc <120%	111.3% (105.6, 114.7)%
Body	D2cc <130%	129.9% (126.9, 130.0)%

**TABLE 2:** Dose objectives and results for 1320 cGy total dose plans (165 cGy per fraction) generated using treatment planning script. Median values with minimum and maximum in parentheses. Objectives defined as a range are primary–secondary goals.

Structure	Dose Objective	Result
PTV	V100% > 90‐95%	95.0% (93.1, 95.2)%
	D98% > 85‐90%	88.7% (85.9, 90.3)%
	Dmean <110%	108.8% (107.1, 110.4)%
Lungs	Dmean <900‐800 cGy	798 cGy (772, 811) cGy
	D0.03cc <120%	111.1% (109.0, 116.4)%
Left kidney	Dmean <1100 cGy	1059cGy (1047, 1067) cGy
	D0.03cc <120%	106.7% (104.7, 110.3)%
Right kidney	Dmean <1100 cGy	1064 cGy (1051, 1081) cGy
	D0.03cc <120%	107.7% (105.6, 108.9)%
Body	D2cc <130%	128.7% (125.7, 130.0)%

**FIG. 7:**
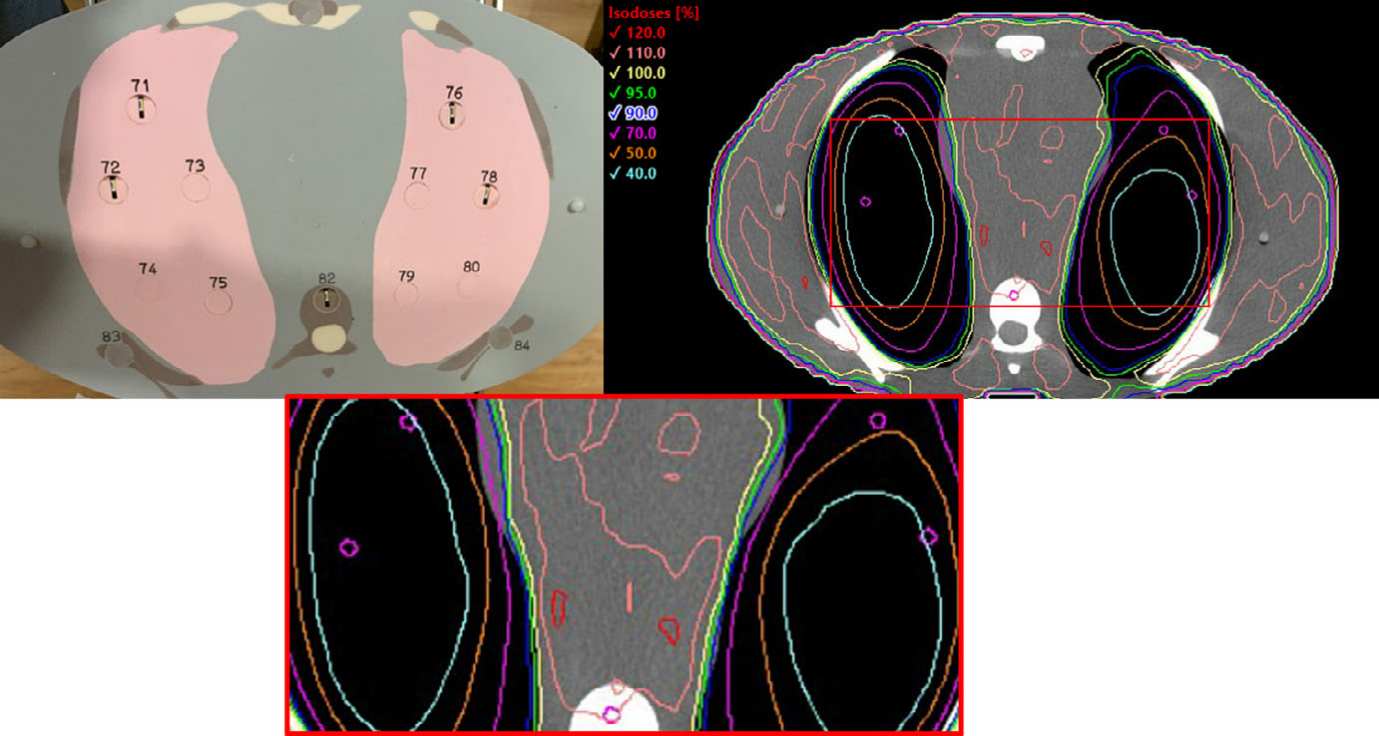
Illustration demonstrating physical location of OSLDs on the CIRS anthropomorphic phantom and contours placed on the treatment planning system to obtain planned dose (purple contours) for one slice. Four OSLDs located in the lungs (plug number 71, 72, 76, and 78) and one OSLD located in the vertebral body (plug number 82). Treatment plan to 1320 cGy in 8 fractions.

**FIG. 8:**
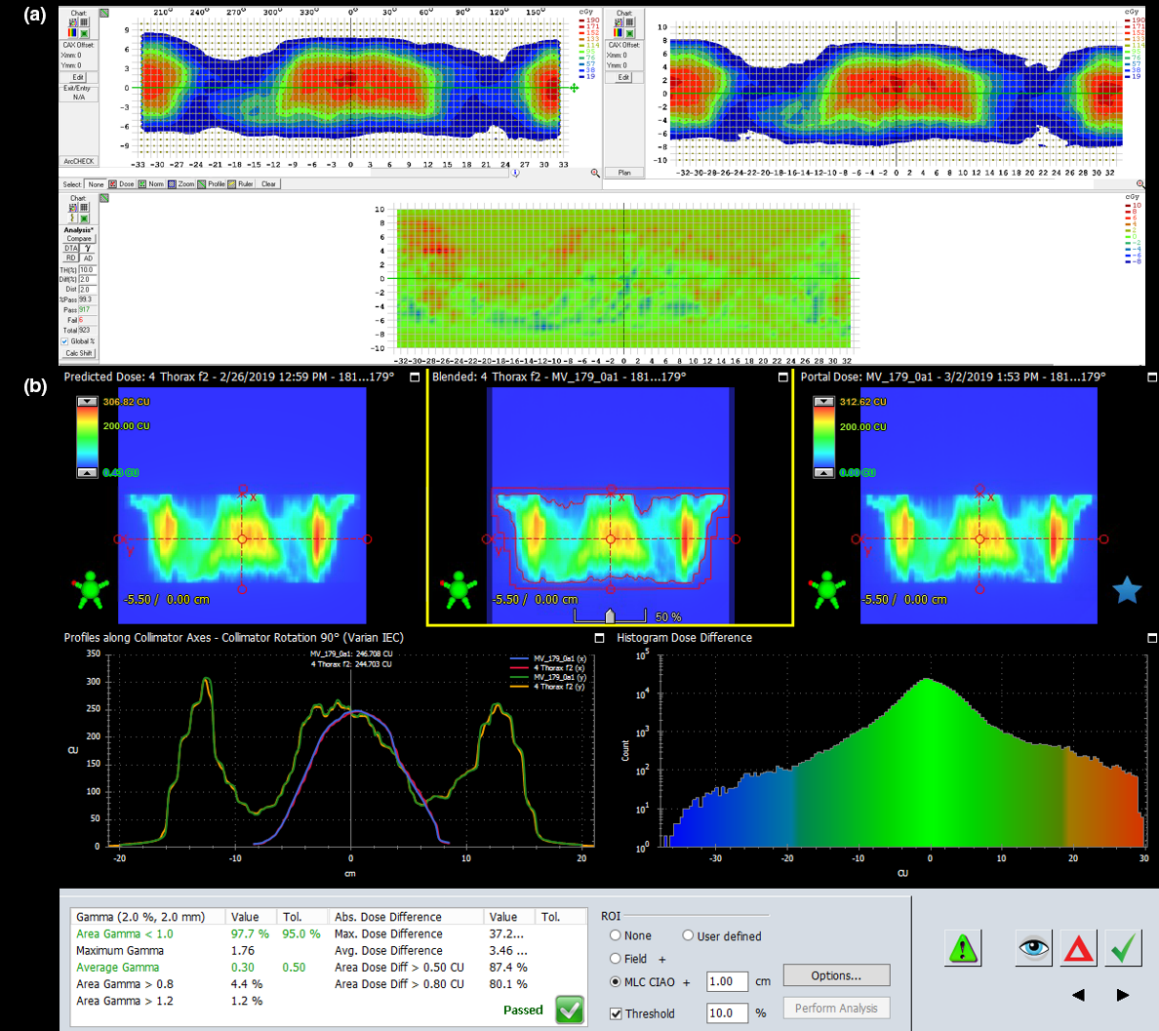
Patient‐specific QA analysis of one chest isocenter field. (a) ArcCheck: 99.3% passing rate at 2%/2mm gamma. (b) Varian Portal Dosimetry: 97.7% passing rate at 2%/2mm gamma.

The normalized dose deviation for all OSLD measurements was less than 4% for each individual OSLD. Individual values are presented in Table 3. Figure 7 illustrates the location of the OSLDs in the anthropomorphic phantom as well as the location taken in the treatment planning system to obtain treatment plan dose. Patient‐specific QA for this plan obtained passing rate over 95% for each individual field and the areas of field overlap using a gamma criterion of 2% at 2 mm. The passing rate was >95% for all three modalities of patient‐specific QA employed. Figure 8 presents the analysis using ArCheck and Portal Dosimetry for one of the chest isocenter fields.

**TABLE 3:** Normalized dose deviation between treatment plan system dose and OLSD measured dose. Differences were normalized using the prescribed dose (165 cGy per fraction).

Location	TPS dose (cGy)	OSLD dose (cGy)	Absolute dose difference (cGy)	Normalized dose deviation (%)
Pelvic bone	185.0	184.6	−0.4	−0.3
Pelvic bone	183.3	186.4	3.1	1.9
Kidney R	123.4	116.9	−6.5	−3.9
Kidney L	103.3	103.8	0.5	0.3
Lung	87.9	83.1	−4.8	−2.9
Lung	66.5	63. 7	−2.8	−1.7
Lung	65.6	62.2	−3.4	−2.1
Lung	58.7	52.5	−6.2	−3.8
Vertebral body	182.3	186.8	4.5	2.7
Vertebral body	182.6	187.6	5.0	3.0
Brain	179.7	178.4	−1.3	−0.8
Brain	185.1	180.6	−4.5	−2.7

**FIG. 9:**
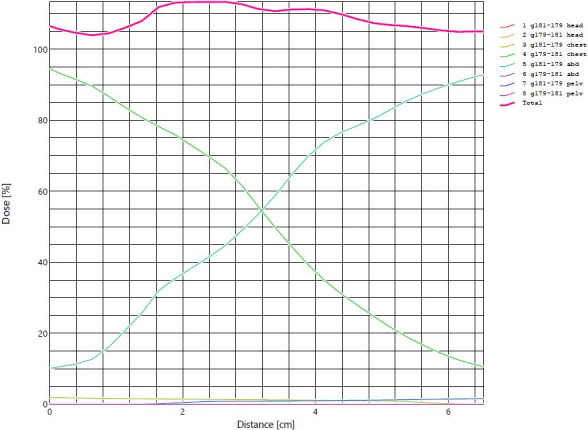
Dose profile at an area of field overlap between chest and abdomen demonstrating linear and smooth feathering between the two fields contributing to the total dose in the area with no abrupt change in field weight within the overlap region.

Our plan uncertainty analysis revealed that the increase in the mean dose to the lungs was always below 3% for all plan uncertainties evaluated with the exception of a 10 mm lateral shift that resulted in a 4.3% mean lung dose increase. Regarding PTV coverage (V100%), our plan uncertainty analysis revealed that the decrease in coverage was below 3% for all scenarios analyzed. After analyzing dose profiles at field junctions we found a dose increase/decrease of about 20% per cm when uncertainty shifts were applied to a single isocenter in the longitudinal direction. This amount was expected considering the use of the auto‐feathering tool in Eclipse and the overlap distance of 5.3 cm. However, there is some variability in areas mostly located at higher dose heterogeneity locations or around high/low‐dose interfaces. Figure 9 includes an example of auto‐feathering at one of the dose junctions.

## 4 DISCUSSION

Our results demonstrate that automating VMAT‐based treatment planning for TBI can reduce treatment planning time, increase consistency in isocenter placement, and decrease variability on field configuration, field matching and field overlap. All the plans generated using this approach met our expected primary dosimetry goals. Additionally, standardization with regard to isocenter placement, and particularly isocenter shifts, can prevent errors and streamline the process for treatment delivery. Furthermore, automation can facilitate the optimization tasks by providing dosimetrists with automated upload of optimization templates. Our in‐vivo end‐to‐end dosimetry delivery to a phantom using multiple QA approaches and OSLDs inside key locations demonstrated that plans are deliverable and dosimetry was accurate using this approach, including areas of heterogeneity, such as the lungs and bone. Our plan uncertainty analysis demonstrated that global shifts (same shift for all isocenters) do not produce large dose deviations as long as they are maintained within 10 mm. The largest uncertainties will occur if the planned longitudinal distance between isocenters is not maintained at treatment. For this reason, we have developed an in‐house software to assist with our image‐guided radiation therapy (IGRT) approach. While a full description of this tool is out of the scope of this report, in brief, the software will prompt therapists to acquire IGRT at three locations (head, chest, and pelvis isocenters) and will calculate an optimal global shift based on the desired shifts at each location. Using this approach, we guarantee that the distance between all isocenters remains constant. The software calculates the residuals between the global shift and the individual shifts desired at each location and will trigger an inspection of setup if established thresholds are not met. Based on our plan uncertainty analysis, thresholds were established at 5 mm for the chest isocenter and 10 mm everywhere else.

The plans presented in this study were obtained with only one optimization and calculation iteration in order to demonstrate that an automated approach is feasible if properly constructed. However, it would be possible to manually adjust the optimization objectives based on experience or to reoptimize to improve the treatment plan. This may actually be necessary if a particular case with a challenging anatomy is presented or if coverage/sparing in a particular area is desired. In any case, a workflow such as the one presented in this study should support standardization and robustness, and provide a good starting point. Additionally, an automatic tool like the one presented here has the potential to include options for additional OARs sparing or simultaneous integrated boost (SIB) regimens beside the two current regimens. There is a clear interest in the radiation oncology community to provide solutions to some of the common problems of TBI, such as organ sparing, lack of or limited imaging capabilities, dosimetric uncertainties, the need to manufacture compensators/blocks and patient comfort. Gruen et al.^22^ reported their initial use of Tomotherapy for VMAT‐based TBI treatments on ten patients treated to 12 Gy with 2 Gy per fraction. The lungs mean dose for this series was 9.14 Gy and no grade 3–4 toxicities were observed. Springer et al.^26^ reported the use of VMAT‐based TBI on a linear accelerator on seven patients. None of the patients reported severe lung toxicities and the authors were able to decrease the dose to the kidneys for patients with renal comorbidities to 7–8 Gy. Tas et al.^25^ reported a VMAT‐TBI technique used to treat 30 patients. The mean dose to the lungs was 9.7 Gy, and the mean dose to the kidneys was 9.6 Gy. Grade 3 toxicity or higher was not observed for any of the treated patients (mean follow‐up of 18 months). Ouyang et al.^27^ reported no pulmonary toxicity for eight patients treated with VMAT‐based TBI on a linac with a mean dose to the lungs of 8 Gy. They introduced a novel rotational immobilization system for this type of treatment. Along those same lines, other institutions are reporting their efforts to develop accurate immobilization for these challenging treatments such as Mancosu et al.^29^ for VMAT‐based TMI treatments and Losert et al.^30^ for linac‐based VMAT‐TBI.

Among the different options available to build this automatic planning tool, we decided on fields collimated at 90° that could keep the X jaw field size at 15 cm except from the most superior arc to allow for flash, and the most inferior arc to allow the optimizer to properly overlap with the lower body 3D base plan. This allows the MLCs from each bank to reach the full extent of the field opening giving the optimizer higher flexibility compared to scenarios where MLCs cannot reach the end of the opposite bank due to larger field sizes. Two fields per isocenter with one field covering superiorly to the isocenter and a second one covering inferiorly (with a 2 cm overlap) proved useful to create MLC‐based island blocks for the lungs and the kidneys. The script is able to adjust that overlap for the chest isocenter fields based on the distance between the chest isocenter and the user origin (Fig. 3). Additionally, for the purpose of this work we kept the overlap of fields not sharing an isocenter to 5.3 cm. This should provide overlap of the diverging beams up to 19 cm away from the isocenter and that sufficed to obtain good coverage for the patient sample used here. However, that overlap can be increased if a challenging case requires it with the only drawback of an increased X jaw field size. We are currently updating the script to directly increase the jaw field if required based on patient geometry for prospective cases.

Our approach for setting up the automatic treatment planning used similar principles as the ones presented by some studies discussed earlier.^24^, ^25^, ^26^ The main contribution from our manuscript is to advance the standardization, reproducibility, and automation of treatment planning for VMAT‐based TBI. Springer et al.^25^ provide a significant advancement in the use of VMAT for TBI. However, the technique for planning was variable among the seven patients with the number of isocenters between 9 and 15 and a different number of fields, full and partial arcs and both longitudinal and lateral shifts. Symons et al.^24^ described a robust technique for the upper body VMAT plan, but the combined lower body plan was not reported and the process required several sequential optimizations to obtain the final treatment plan. In our manuscript, we report a fully standardized and reproducible technique with the same number of isocenters, same shifts between upper body isocenters and consistent overlap of fields and jaw settings to create MLC driven island blocks. In addition, we have described the use of ESAPI to automate most of the process so only an estimate of 10–15 min of manual tasks are required (5 min for OAR contouring plus 5–10 min for field weight adjustment of the lower body plan).

Dose rate is a subject of controversy with regard to TBI. We selected an energy of 6 MV photons and a nominal maximum dose rate of 600 MU/min for this work. For conventional treatments it is recommended to keep the dose rate below 20 cGy/min or even 10 cGy/min to minimize lung toxicities.^2^, ^10^, ^31^ However, there is not an accurate way to compare the influence of dose rates between modulated and nonmodulated treatments. For open field at‐distance treatments, the lungs will receive the dose uniformly, while on modulated treatments there are regions of the lungs receiving doses as low as 40 cGy per fraction (Fig. 7) with a gradual increase as we reach the external boundary of the lungs. Also, the complete volume of the lungs is never irradiated at the same time, and the mean dose can be decreased significantly. Currently, there is no clear evidence as to how dose rates may affect modulated TBI treatments where the dose to the lungs is significantly decreased. Tas et al.^25^ reported in their study a mean instantaneous dose rate of 250 MU/min (range 50–600 MU/min) and did not encounter any grade 3 or higher lung toxicity for any patient (n = 30). The dose rate reported by Tas et al. is practically identical to the one presented in this study. Despite using a 600 MU/min maximum nominal dose rate, during delivery to our phantom the instantaneous dose rate was observed to range between 200 and 250 MU/min during most of the delivery, with some lows of 50–100 MU/min, and highs of up to 400–500 MU/min. In spite of this, the automatic treatment planning workflow presented in this study can be employed with a different nominal dose rate or beam energy.

After treatment is completed and signed‐off the tool prints out a summary report of the treatment. From our experience with conventional TBI using lateral beams, a total beam‐on time of about 20 min is common. However, setup of the patient at distance, OSLD placement, spoiler placement, compensation with solid water for the head, compensation with rice bags between the legs and checklist procedure adds about 30–40 min for the first side, and another 10 min to rotate the patient and verify setup for the opposed field. Using the proposed VMAT‐TBI approach the beam‐on time per treatment is about 10 min. While VMAT‐TBI eliminates several components pertaining only to the conventional approach, the addition of imaging, need for shifts and patient orientation shift are also time‐consuming and have to be executed cautiously. Our initial experience through dry‐runs and test of end‐to‐end process indicates that a total treatment time between 1 hr and 1 hr and 15 min should be expected for VMAT‐TBI and that remains within the same time range of the conventional approach.

Our study has several limitations. First, the CT datasets employed were acquired on a different position compared to the one expected for a real VMAT‐based TBI program (currently under development at our institution). In the CT datasets used the patient was positioned with forearms crossed over the chest, arms at the lungs level, and lower extremity separation similar to pelvis or shoulder separation. This positioning differs from our defined CT simulation positioning for VMAT‐TBI with legs tighter together, arms at patient side and thermoplastic immobilization. While this is a limitation, our scripted treatment planning approach was able to provide high‐quality plans even using the less favorable positioning for VMAT planning. Second, while the five cases explored here include patients with diverse body habitus, this is a very limited number and patients with even larger anatomic variation might pose a challenge at presentation. Finally, some information that the script uses for the field arrangement is specific to our institution. As an example, the script will always maximize the body region covered by the upper body VMAT plan. The longitudinal (craniocaudal) distance that is allowed is based on the indexing of the immobilization device to be employed at our institution and the longitudinal travel limit of the couch (with a margin to prevent reaching maximum travel). However, this does not conflict with the applicability of our field arrangement to other immobilization or setup scenarios.

## 5 CONCLUSIONS

In our study, we have demonstrated that VMAT‐based TBI treatment planning can be automated using scripting. Scripting can produce high‐quality plans based on predefined dose objectives and can decrease planning time by automatic target and optimization contours generation, plan creation, field and isocenter generation, and optimization objectives setup. Additionally, a robust and standardized planning approach that accounts for couch longitudinal limits and immobilization facilitates treatment delivery ensuring consistent shifts and isocenter placement.

## 6 PREVIOUS PUBLICATION OF MANUSCRIPT TEXT OR DATA

Part of the material presented in this manuscript was previously submitted and presented at the 2019 AAPM annual meeting and the 2019 ASTRO annual meeting. The submitted abstracts are published in the conference proceedings:

1. Teruel, J. R.; Taneja, S.; McCarthy, A.; Galavis, P.; Malin, M.; Osterman, S.; Gerber, N. K.; Barbee, D.; Hitchen, C. Robust VMAT‐based Total Body Irradiation (TBI) Treatment Planning Assisted by Eclipse Scripting. International journal of radiation oncology biology physics. 2019:105(1):E788‐E789.
2. Teruel, J; Taneja, S; Galavis, P; Osterman, K; Malin, M; Gerber, N; Hitchen, C; Barbee, D. VMAT‐based total body irradiation treatment plans with eclipse scripting for field configuration: A dosimetric evaluation. Medical physics. 2019. https://doi.org/10.1002/mp.13589.

## CONFLICT OF INTEREST

Honorarium from Varian Medical Systems (Jose Teruel).

## Supporting information

Supplementary Material
