# Supplementary material for: Automatic treatment planning for VMAT‐based total body irradiation using Eclipse scripting
Source: J Appl Clin Med Phys. 2021 Feb 10;22(3):119–30. doi: 10.1002/acm2.13189 (PMC7984467; doi:10.1002/acm2.13189)

**Supplemental Figure 1.** Flow chart demonstrating treatment planning steps. Steps presented in green background were completely automated using the approach described in this manuscript. All automated steps presented are completed in less than 10 seconds with the exception of the step marked with *.

Steps presented in light green background and dashed arrows were not employed for the plans presented in this manuscript, but will probably be necessary for prospective cases to improve dose objectives. These steps are expected to be manual, but are susceptible for automation after several prospective cases are planned.

*Optimization and calculation of VMAT plan: This step can be run automatically but it does not result in time savings for the process.


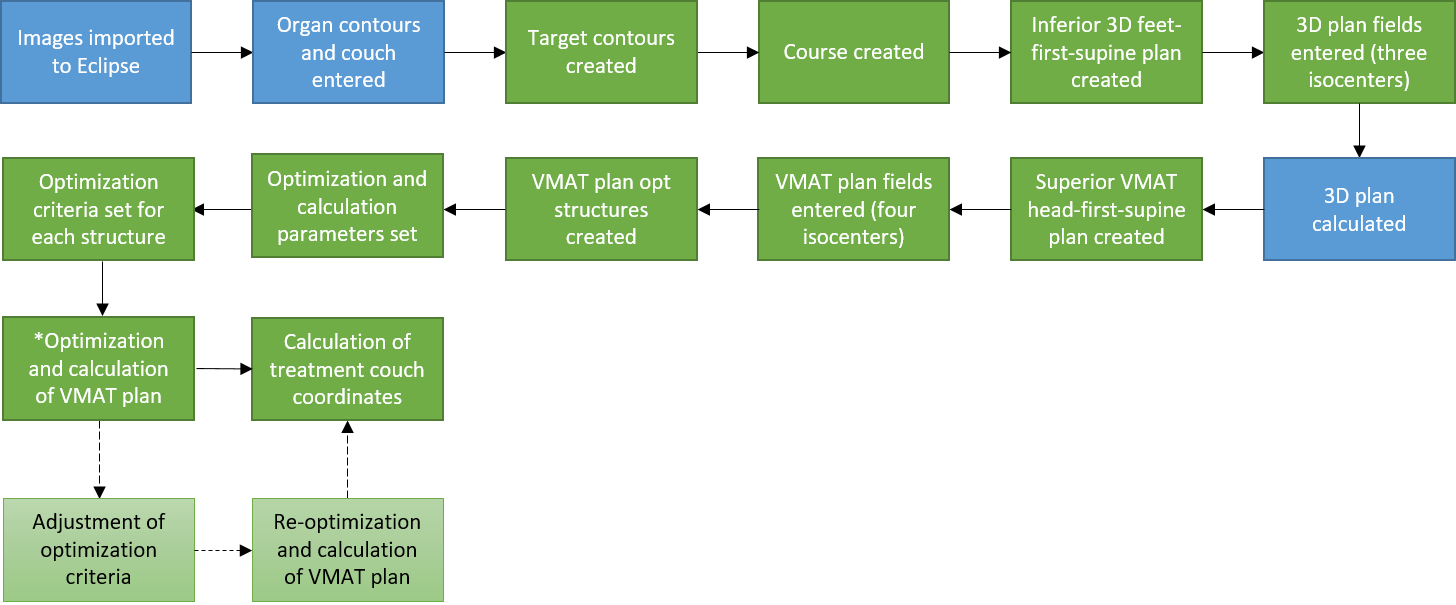

Supplement: Supplementary file 1 — Supplementary Material [file ACM2-22-119-s001.docx]
